# Sustainable high performance computing in the UK: a position paper on public investment, accountability, and the path to net zero

**DOI:** 10.1007/s11227-026-08946-z

**Published:** 2026-10-22

**Authors:** Sylvain Laizet, Sudhir Rama Murthy, Pelin Demirel, Peter Childs, Evangelos Danopoulos, John Aston, Nick Brown, Michèle Weiland

**Affiliations:** 1 Department of Aeronautics, Imperial College London, London, UK; 2 Dyson School of Design Engineering, Imperial College London, London, UK; 3 Department of Pure Mathematics and Mathematical Statistics, University of Cambridge, Cambridge, UK; 4 EPCC, University of Edinburgh, Edinburgh, UK

**Keywords:** High performance computing, Sustainability, Accountability, Artificial intelligence

## Abstract

The UK invests significantly in High-Performance Computing (HPC), yet has no coherent national strategy that addresses sustainability, accountability, or long-term infrastructure planning. This paper proposes and promotes three key messages: first, that the return on public investment cannot be assessed without consistent public reporting on utilisation, research outputs, and cost-effectiveness for all publicly funded HPC systems; second, that publicly funded HPC systems should be subject to mandatory, standardised reporting on energy consumption, carbon emissions, with information on what happens when a system is decommissioned; and third, that the conflation of AI and HPC in UK policy is a strategic error. Instead, HPC is the underpinning infrastructure, and AI is one of many types of applications using that infrastructure, alongside numerical modelling and simulation. Treating AI and HPC as separate, competing workloads risks under-serving the wider research community that depends upon that shared infrastructure, resulting in a weakening rather than strengthening of the UK’s national compute capability.

## Definitions and scope

The UK government has used the terms HPC, compute, AI infrastructure, and data centre interchangeably and inconsistently in policy documents, funding announcements, and strategy papers over the past several years. As a result, it is difficult to compare investment figures and to evaluate whether stated commitments have been met. The definitions in this paper are therefore foundational to the arguments that follow and call on government and funders to adopt consistent terminology in future policy and procurement documentation.

### Key definitions

The following definitions are used throughout this paper.

#### High performance computing (HPC)

HPC refers to computing systems designed to perform calculations at speeds and scales significantly higher than those of standard desktop or server hardware. HPC systems typically consist of clusters of compute nodes connected by high-speed interconnects, with shared parallel storage systems. Compute nodes in HPC systems are typically built around CPUs, GPUs, or a combination of both. Their defining features are the capacity for massively parallel computation, low-latency inter-node communication, and high-throughput I/O. These characteristics are architectural and performance-based, and do not depend on ownership or funding model: institutional clusters, dedicated mission systems such as those operated by national weather services, and industrial systems all qualify as HPC.

#### HPC facilities and data centres

HPC facilities are specialist buildings or dedicated sections of data centres designed to house and operate HPC systems. They encompass not only the computer hardware itself, but also the power, cooling, networking, and storage infrastructure. HPC facilities range from high-density, liquid-cooled environments required for the most energy-intensive modern hardware, to air-cooled facilities with traditional computer room air conditioning units. The term data centre is used in this paper to describe the physical facility, while HPC facilities refer to the combination of that infrastructure and the HPC systems it hosts. Funding models differ: national (or regional) HPC facilities are typically funded through research councils, whereas general-purpose data centres may be commercially or institutionally funded.

#### Cloud computing

Cloud computing is a computing environment in which compute, storage, and networking resources are delivered as virtualised services on-demand over a network, rather than as hardware owned and operated directly by the user. They are typically built on large pools of servers housed in large data centres, with resources allocated flexibly. Their defining features are flexibility, self-service provisioning, and consumption-based billing, which allow capacity to be acquired or released almost instantly without capital investment. In the UK research context, cloud systems are increasingly used as a hosting platform for data services and interactive analysis, and for workloads requiring heavy CPU/GPU power or large memory capacity (beyond what is on offer via traditional HPC facilities).

#### Server farms and hyperscalers

Commercial cloud providers, most notably Amazon Web Services (AWS), Microsoft Azure, Oracle Cloud Infrastructure, Alibaba Cloud, and Google Cloud, operate large-scale data centre infrastructure at a scale that dwarfs any national research HPC programme. Known as hyperscalers, the largest cloud providers operate massive, global data centres with large, flexible capacity designed to support virtually every type of organisation, from large enterprises, governments, and tech companies to startups, and healthcare providers. Essentially, any organisation that runs software or stores data is likely to be a customer of at least one hyperscaler. Note that commercial cloud is outside the scope of this paper, which focuses on dedicated, publicly funded HPC systems.

#### AI versus HPC

Artificial intelligence (AI) is one workload amongst many that run on HPC infrastructure, typically on GPUs. Consequently, AI is an application domain that makes use of HPC systems. In the UK, however, AI and HPC are frequently conflated in public discourse and policy, with concrete consequences for how national computing resources are planned and allocated (note that few other countries are following the same strategy). A notable example is the strategic decision to commission HPC systems that are exclusively dedicated to AI workloads. This separation reflects funding and programmatic boundaries rather than hardware capability: non-AI workloads, many of which are technically compatible with the same hardware, are excluded from these systems by policy rather than by technical constraint.

This separation raises questions about resource efficiency. GPU systems procured for AI retain substantial double-precision capability. Such systems can therefore in principle support a broad range of scientific computing tasks, from climate modelling to computational fluid dynamics. Restricting such systems to a single application domain narrows the workload mix available to the scheduler, and hence the headroom available to sustain high occupancy. This matters because the marginal cost of idle capacity on large systems is high because idle compute nodes draw approximately 50% of fully loaded power, interconnect switches draw essentially constant power irrespective of load, and where embodied emissions dominate the lifetime footprint, efficiency depends on extracting the maximum output per node-hour over the system’s lifetime [1].

It is important to highlight that this is an argument about scheduling flexibility rather than an empirical claim about any particular facility. To the best of our knowledge, operational utilisation statistics for the UK’s AI-dedicated national systems are not currently published, and the effect of domain restriction on achieved utilisation therefore cannot be quantified from public data. We firmly believe that the publication of utilisation and energy statistics for these systems as a prerequisite for evidence-based capacity planning, consistent with the case for standardised operational reporting developed in this paper.

#### Sustainability

In this paper, sustainability refers to minimisation of the environmental impact of HPC facilities and operations throughout their full lifecycle. This includes operational energy consumption and associated carbon emissions; embodied carbon in hardware manufacturing, transport, and installation; hardware end-of-life management, including recycling and repurposing; and water consumption associated with cooling.

#### Users of HPC

The users of publicly funded HPC in the UK span a broad range: academic researchers in universities across all disciplines; public sector organisations (such as the Met Office, even if some of its compute infrastructure is now largely cloud-hosted [2] they still heavily rely on publicly funded HPC systems); and, to a lesser extent, industrial R&D teams. This paper focuses primarily on academic and publicly funded use.

### Scope of this paper

This paper is concerned with publicly funded HPC systems in the UK, which are used primarily for research. These include national facilities, regionally funded (medium size) systems, and dedicated AI compute investments made through public funding bodies. Within the technical definition given in Sect. 1.1, the systems considered here are those that are shared, multi-user, and nationally or regionally funded. Institutional Tier 3 systems, dedicated operational systems, and industrial HPC are therefore outside the scope of this paper, despite the fact that they meet the same architectural definition.

Sustainability and accountability frameworks governing these systems are discussed, alongside government strategy and investment decisions. Comparisons are also drawn to approaches taken in other countries, all within the broader context of the UK’s path to net zero. Enterprise IT infrastructure,^1^ commercial cloud services, and private-sector data centres fall outside the scope of this paper.

It is important to point out that although our analysis is grounded in the UK context, the questions raised in this paper are global. Every research-intensive nation is grappling with how to govern the growing energy footprint of scientific computing, how to ensure accountability for public compute investments, and how to balance the current political emphasis on AI against the continuing needs of the wider scientific computing community.

All factual claims in this paper concerning systems, investments, procurement decisions, and timelines reflect publicly available information as of September 2026. This is a fast-moving policy area and several of the commitments discussed here are expected to develop further. We therefore distinguish throughout between what is confirmed (announced and contracted, or operational), planned (announced with a stated timeline but not yet delivered), expected (indicated by funders or operators, but not formally committed), and proposed (advanced in policy documents without associated funding). Readers should treat forward-looking dates with caution.

### Article type & objective

This is a community position paper, with contributions from researchers who operate, use, and develop software for UK national HPC infrastructure. It argues a case rather than reporting an experiment: it is neither an empirical research article generating new primary data nor a systematic review. Its purpose is to consolidate the evidence currently dispersed in government strategy and policy announcements, facility documentation, and funder publications into an argued position and to convert that position into requirements that can be written into procurement and funding conditions.

The question the paper addresses is: what reporting, procurement, and governance conditions would allow the return on UK public investment in HPC, including its environmental cost and sustainability, to be properly demonstrated rather than asserted?

### Sources and method

The paper draws on five categories of public source: (i) UK government publications and press releases, principally from DSIT, HM Treasury, and the UK Atomic Energy Authority; (ii) publications by UKRI and research councils, mainly the ARCHER2 evaluation [3]; (iii) operational documentation, technical specifications, quarterly service reports and peer-reviewed operator case studies from EPCC, DiRAC, JASMIN, the Hartree Centre and the AIRR sites; (iv) international programme documentation from EuroHPC, the US DOE and RIKEN R-CCS; and (v) independent ranking and analytical sources, primarily TOP500 [14] and [15]. Unpublished, confidential, or commercially restricted data were not used, and no Freedom of Information requests were made.

Systems are included where they are located in the UK, substantially publicly funded, and intended primarily for research use, consistent with the scope in Sect. 1.2. Comparator countries were selected on the basis of comparable research intensity together with the existence of an articulated national HPC strategy, yielding the USA, the European Union via EuroHPC, Japan, China, France, Germany, and the Nordic countries.

The method is documentary analysis and structured comparison rather than statistical inference. Claims are checked across at least two independent sources where possible. Interpretive judgements, particularly the assessment of the dual-track strategy in Sect. 7 and the recommendations in Sect. 8, are the considered position of the author group, drawing on operational experience. We identify these as positions rather than findings.

We would add that the difficulty of assembling a consistent evidence base is not incidental to the paper but is one of its findings. For several UK systems within scope, energy consumption, utilisation rates, and end-of-life plans are not in the public domain, and for the most recent investments the reporting obligations, if any, have not been published. These are precisely the issues Sects. 4 and 5 seek to resolve.

The paper is organised as follows. Section 2 discusses the role of HPC in research, with a focus on the UK landscape. Section 3 provides international comparisons, examining government HPC strategies in a number of countries of relevance. Section 4 addresses the sustainability and environmental impact of HPC, and Sect. 5 considers accountability, transparency, and the return on public investment. Section 6 discusses skills, training, and user support, and Sect. 7 examines the relationship between AI investment and HPC. Section 8 sets out our recommendations, and Sect. 9 concludes the paper.

## The role of HPC in research

HPC enables researchers to run complex simulations, process large datasets, and solve computationally intensive problems that are not feasible on standard computing hardware. These problems are intractable on conventional systems for several reasons: the computation may take an impractical amount of time to complete, the simulation may exceed the memory capacity of a conventional system, the volume of output data may be too large to store or transfer using standard infrastructure, or the solution method may require levels of numerical precision and parallelism that are only achievable at scale. Access to HPC is therefore a critical component of a modern research infrastructure, enabling universities and research institutions to conduct work at the frontier of scientific discovery.

### HPC as a research infrastructure in the UK

HPC has been central to computational science since the 1970 s and 1980 s, when vector-based supercomputers enabled the first practical large-scale numerical simulations. In the UK, investment in national HPC infrastructure began to formalise in the 1990 s with the establishment of centres such as CSAR and EPCC. CSAR (Computer Services for Academic Research) was a UK national supercomputing service operated by the University of Manchester from 1998 to 2006. EPCC (formerly the Edinburgh Parallel Computing Centre) is a supercomputing centre based at the University of Edinburgh, created in 1990 with funding from the UK government, with a stated mission to accelerate the effective exploitation of novel computing throughout industry, academia, and commerce. The creation of EPSRC in 1994 consolidated HPC investment within a single research council framework. EPSRC is the main UK government agency responsible for funding research and training in engineering and physical sciences and is part of UK Research and Innovation (UKRI).^2^

EPCC has hosted almost all of the UK’s national HPC services, including the first UK national supercomputing service, a Cray T3D with 512 CPUs, which was operational between 1994 and 1999. During that time, CSAR provided HPC facilities and expertise to UK researchers from 1998 to 2006 with two SGI machines, comprising 512 CPUs, called Newton and Green. From 2002 to 2010, HPCx (an IBM Power5 system with 1280 cores, later upgraded to 2560 cores) was the UK national service run by EPCC, but with the system located in Daresbury. More recently, the UK national services were all hosted by EPCC and located in Edinburgh: HECToR (Cray XT4/XE6, 2007-2014); ARCHER (Cray XC30, 2013-2021), and ARCHER2 (HPE Cray EX, 2021-present). All of these CRAY systems are CPU-based machines.

Today, HPC enables research across a wide range of disciplines. These include atmospheric and climate modelling, structural biology and genomics, materials science and condensed matter physics, engineering simulation, computational chemistry and drug discovery, astrophysics and cosmology, and computational social science.

Thanks to HPC, researchers can tackle a broad array of scientific problems and investigate them in much greater detail and precision than would otherwise be feasible. HPC dramatically reduces the cost and risk of scientific exploration, where complex simulations allow researchers and innovators to test a large number of scenarios computationally. Ultimately, this results in the physical experiments that are eventually carried out being much more targeted and impactful. For example, a research team no longer needs to synthesise and test hundreds of material samples to identify promising candidates. Instead, it can narrow the field through simulation and invest experimental resources where they will yield the greatest return.

The academic impact of HPC-based work is substantial and measurable. According to its end of life report, ARCHER2 alone has supported the publication of more than 2,100 articles covering 20 different fields of research. These have been cited more than 27,000 times, with an average field-weighted citation index of 2.6, which means that those articles receive, on average, 2.6 times the citation rate expected for comparable publications [3].

In cardiovascular medicine, for instance, researchers at Imperial College London are using HPC to build personalised digital twin simulations of patients’ hearts, allowing clinicians to virtually test different treatment options before intervening. In antimicrobial research, researchers at the University of Oxford have produced some of the most detailed computational models of bacterial outer membranes ever created, resulting in generating new insights into how antibiotics might overcome bacterial defences at a time when antibiotic-resistant infections contribute to over 40,000 deaths annually in the UK alone. In renewable energy, researchers at the University of Plymouth are using HPC to develop new wave modelling techniques that reduce the time and cost of designing floating offshore wind turbines to survive harsh marine environments. In aerospace, simulations on ARCHER2 have enabled researchers at Newcastle University and the University of Cambridge to model hydrogen combustion in turbulent aero-engine conditions in collaboration with Rolls-Royce, work that could reduce aviation’s in-flight climate impact by up to 75%. UK researchers using the NEMO ocean modelling framework have been able to produce high-resolution simulations of ocean circulation, sea level change, and marine ecosystem dynamics that directly inform the Met Office’s operational weather forecasting system and contribute to international climate assessments [3].

The HPC skills pipeline is also a significant source of economic and societal value. More than 2,650 students and postdoctoral researchers have gained hands-on experience on ARCHER2. Of those who remain in the UK, approximately 44% go on to careers in industry, transferring advanced computational skills into sectors ranging from pharmaceuticals and aerospace to financial services and energy. Independent evaluation estimates that the total economic benefits enabled by ARCHER2 amount to £4.3 billion over its lifetime, representing a return of £8.3 for every £1 of public money invested [3].

### The current UK HPC landscape

#### National facility: ARCHER2

ARCHER2 is the current UK National Supercomputing Service, hosted at EPCC (University of Edinburgh) and mainly funded by EPSRC and NERC through UKRI. It was commissioned in 2020 and is based on a Cray EX architecture with 750,080 CPU cores (AMD EPYC processors) and a Slingshot high-speed interconnect. ARCHER2 is a CPU-based system that focuses on simulation and modelling workloads. Access is open to UK researchers via various routes, and as of September 2025, ARCHER2 had nearly 9400 registered users. Of these around 5,750 were active which is defined as users who have run jobs on ARCHER2 between 2021 and 2025 [3]. ARCHER2 is scheduled for decommissioning in November 2026, approximately five years after it became operational. A new national facility was announced in 2025 with up to £750 million for the project, but it will only be operational for research in 2028, leaving a gap of more than a year without any national facility [4].

#### Specialist national facilities and initiatives

DiRAC is a Science and Technology Facilities Council (STFC)-funded HPC facility serving the particle physics, astrophysics, and cosmology communities [5]. It is a distributed service across multiple UK universities. JASMIN, operated by the STFC’s Scientific Computing Department and the Centre for Environmental Data Analysis (CEDA), provides storage and computing infrastructure specifically for environmental and climate science [6]. The Hartree National Centre for Digital Innovation (HNCDI) is a collaborative programme with IBM that enables businesses and public sector organisations to explore and adopt advanced digital technologies, including supercomputing, data analytics, AI and quantum computing [7]. The HNCDI launched Mary Coombs in October 2025, a new AI-focused supercomputer named in honour of the UK’s first female commercial programmer.^3^

#### Medium size systems

Several medium-sized HPC systems, which are smaller in scale than national facilities, have been funded through UKRI and hosted at UK universities. These have historically been referred to as Tier 2 systems within a tiered classification framework. Tier 0 corresponds to large international facilities (some of which have exascale capabilities, and as of 2026 Europe has only one exascale system which is called JUPITER). Tier 1 are national facilities (like ARCHER2), Tier 2 are regional supercomputers (where region can be geographical or application domain) and Tier 3 are typically smaller university-level systems. The newly established National Compute Resources (NCR) programme is expected to broaden access for the research community by deploying a mix of next-generation medium size CPU- and GPU-based systems which have been tailored to diverse research needs [8]. To date, three CPU-only NCRs have been announced: Cirrus at the University of Edinburgh with 184,320 AMD CPU cores, Charger at UCL/DataVita with 37,000 Intel Xeon CPU cores, and Isambard 3 at the University of Bristol with 54,720 NVIDIA Grace–Grace cores. Furthermore three GPU-based NCRs have also been funded and these are Baskerville at the University of Birmingham with 200 NVIDIA GPUs, Zenith at the University of Cambridge with 224 AMD GPUs, plus Mary Coombs at Daresbury with 628 NVIDIA GPUs. These six NCRs will be deployed in 2026-2027 [8].

#### AI-dedicated investments

The AI Research Resource (AIRR) is a suite of HPC systems funded by the UK government to address the under-provision of public compute resources for AI users [9]. AIRR currently offers compute access through two supercomputers: Isambard-AI at the University of Bristol and Dawn at the University of Cambridge. Isambard-AI, containing over 5,400 NVIDIA superchips, is currently the UK’s most powerful supercomputer. Dawn, an Intel-powered GPU system, is being updated with a £36 million investment to include AMD’s latest AI chips which will increase its capacity sixfold. The next iteration of Dawn is expected to become operational in 2026. As part of the AI Opportunities Action Plan [10], the government announced in January 2025 that the capacity of AIRR would expand at least 20 times by 2030. In June 2026, the UK government unveiled a £1.1 billion AI Hardware Plan, including £750 million for a new national AI supercomputer (with £400 million earmarked for next-generation chips, part of which supports British startups), plus a new playground global-led investment fund backed by up to £150 million from the British Business Bank. The plan also includes £120 million for an AI Hardware Innovation Programme and £45 million for skills development, with the aim of growing Britain’s domestic chip and AI hardware industry, jobs and national security capabilities [11]. Finally, the UK government is also investing £45 million in a new AI supercomputer called "Sunrise", which will be housed at the UK Atomic Energy Authority’s Culham Campus [12].

### Funding history and assessment

UK investment in HPC has proceeded through a series of discrete, periodically renewed funding cycles. Key recent systems and approximate investments include HECToR (2007-2014, approximately £138 M), ARCHER (2014-2021, approximately £82.5 M) and ARCHER2 (2021-2026, approximately £100 M). These numbers include both capital and operational costs. The most significant recent funding announcement has been the commitment of £750 million for a new national facility, which can be thought of as ARCHER2’s successor, and the investments around AI via AIRR.

Alongside hardware investment, the UK has also committed funding to software development and skills training. Recent examples include the ARCHER/ARCHER2 embedded CSE (eCSE) programme, which provided dedicated funding for specialist software engineers to develop, optimise, and maintain scientific codes for the ARCHER and ARCHER2 platforms [13]. Complementing this, the ExCALIBUR programme (Exascale Computing ALgorithms and Infrastructures Benefiting UK Research) invested £47.5 million from October 2019 to March 2025 to prepare UK simulation codes and numerical methods for the next generation of exascale systems [14]. Furthermore, the £129 million Digital Research Infrastructure (DRI) programme has to date invested heavily in skills and career development for digital Research Technical Professionals (dRTPs). Considering that the next generation of supercomputer will force a step change in execution from CPU only to GPU-accelerated systems, investment in the software is essential to ensure that the UK scientific community can leverage these new investments.

The procurement and deployment of the new national HPC facility have been subject to notable delays, reflecting broader uncertainties in UK research funding policy. The 2024 government change imposed a review period during which long-term commitments to large-scale research infrastructure faced renewed scrutiny. There are growing concerns within the HPC and the wider research community that successive governments have struggled to obtain a clear understanding of the scientific and economic value that national HPC capability delivers.

A structural issue in the history of UK HPC procurement is that, until recently, the contracts for hardware, hosting, and computational science and engineering support were procured separately. An example is HPCx for which the hosting site was Daresbury, the vendor was IBM, and the computational science and engineering support provided by EPCC. This procurement model means that the host did not participate in the procurement process for the national system that it would operate and systems were selected and contracted independently of the constraints of the site’s machine room, power infrastructure, and cooling capacity. This practice has been revised for the next national service, bringing the UK in line with international norms.

Ultimately, to date, HPC funding in the UK has been episodic, reactive, and not sufficiently integrated with sustainability planning. Funding cycles of five to seven years do not support the longer-horizon infrastructure investments, such as modifications to physical data centre builds, cooling systems, and renewable energy contracts, that sustainable HPC operations require.

## International comparisons

It is instructive to contrast against the international context in order to understand the UK’s position in HPC. The term *national capability* is most meaningfully assessed relative to the ambition and investment of peer nations. Without international references there is a risk that UK funding decisions are made in isolation, possibly underestimating the scale of investment required to remain competitive. A useful starting point is to use the number and scale of a country’s supercomputing systems as an indicator of its investment in frontier research and technological positioning. According to the Top500 list^4^ the UK is not in the top 10 countries in the world for the number of HPC systems [15]. Instead, this ranking is dominated by the USA, Japan, Germany, and China.

Another important metric is the per-capita HPC ranking, which is the number of compute resources available per million inhabitants. In 2025, this ranking was dominated by Finland, Switzerland, and the USA [16]. The UK is not in the top 10 of that ranking, whilst France, Germany, Italy, and Spain are. The per-GDP HPC intensity ranking, which measures the ability to convert economic resources into computing power, was also led by Finland and Switzerland in 2025. This ranking is dominated by EU countries, Japan, China, and the USA and once again the UK is not in the top 10 [16]. It should be noted that none of these rankings account for the long-term sustainability of the strategy underpinning HPC investments, which is a much more difficult metric to quantify.

### Government HPC strategies in other countries

#### USA

The USA funds HPC primarily through the Department of Energy (DOE), operating under a long-term national strategy like the Exascale Computing Project, ECP, which ended at the end of 2024. This programme delivered two exascale systems, Frontier (Oak Ridge National Laboratory, operational from 2022) and Aurora (Argonne National Laboratory, operational from 2025). The DOE model benefits from long-term, multi-year funding certainty and deep integration between facility operations, system software development, and application support. The U.S. National AI Research Resource (NAIRR) programme extends this model into AI infrastructure which follows the same strategy as in the UK but at a much larger scale. The DOE national laboratories report energy metrics to DOE and have set internal energy metrics goals. DOE publishes best-practice guidance on energy metrics and energy efficiency for data centres, but this is guidance, not procurement requirements. Recent DOE supercomputer procurements focused on performance, architecture, and mission needs, not explicitly on energy metrics or renewable energy sourcing [17]. In general, the DOE flagship supercomputing programme is currently expanding, driven by AI. However, the broader research funding ecosystem that supports HPC users, particularly through NSF and university grants, is currently under serious pressure.

#### European Union: EuroHPC

The EuroHPC Joint Undertaking (JU) is a co-funding model in which the EU contributes 50% of capital costs and member states provide matching funding [18]. Current EuroHPC systems include LUMI (Finland, powered by 100% renewable hydropower with waste heat recovery), Leonardo (Italy), MareNostrum 5 (Spain), JUPITER (Germany, the first EuroHPC exascale system), and Alice Recoque (France, under deployment). EuroHPC mandates sustainability reporting and has made renewable energy sourcing a formal criterion in site-building decisions. The European Processor Initiative is developing a European-designed HPC processor as part of the EU’s strategic objective of reducing hardware dependency on non-European suppliers. SiPearl, spun off from EPI in 2019 to commercialise the high performance microprocessor technology, has developed the Rhea1 CPU which is deployed in JUPITER’s CPU partition [19].

#### Japan

Japan operates its national HPC strategy through RIKEN’s Centre for Computational Science (R-CCS) [20]. Fugaku (Fujitsu A64FX ARM CPU processors, no GPUs) was the world’s fastest supercomputer from 2020 to 2022 [21]. This reflects Japan’s strategic preference for custom CPU-based architectures over the GPU-accelerated hardware that has come to dominate modern HPC deployments. Japan has also demonstrated a commitment to extended operational lifetimes of its machines. Both the K Computer [22] and Fugaku were planned for approximately eight-year lifetimes which is three years above beyond the five-year lifespan that ARCHER2 will likely enjoy.

#### China

China’s HPC strategy is delivered through a network of National Supercomputing Centres (Wuxi, Tianjin, Guangzhou, Shenzhen and others), funded through national science and technology programmes and increasingly orientated towards technological self-sufficiency. Successive U.S. export controls have forced China to develop its own hardware [23]. It is also difficult to evaluate China’s HPC capabilities as it often does not contribute to the TOP500 list. In June 2026, LineShine was ranked as the fastest exascale supercomputer in the world, the first China-based system to lead the list since Sunway TaihuLight in 2017 [24]. Like Japan, China seems to have pursued a CPU-only architecture at the leading edge, and like Fugaku base their system around the ARM architecture although for reasons of supply chain independence and application balance, with the entire stack from processor to operating system is manufactured domestically. It is notable that LineShine is only number 50 on the Green500 list, a biannual ranking of the world’s supercomputers by energy efficiency [25], compared with El Capitan which is number 28 on that same list and the second most powerful machine in the world as of June 2026.

#### Other National Strategies

France (GENCI) operates a multi-system portfolio with explicit sustainability commitments and a long-term national roadmap. Germany’s Gauss Centre for Supercomputing coordinates three national centres (Jülich Supercomputing Centre [26], Leibniz Supercomputing Centre [27], High-Performance Computing Center Stuttgart [28]) under a coherent funding framework. The Nordic countries, particularly Finland and Sweden, have achieved exemplary sustainability outcomes through co-location with renewable energy sources and waste heat recovery systems, where LUMI at CSC in Finland [29] is the most prominent example.

In particular, most of these systems now follow a pattern of regular incremental upgrades rather than full replacements on five- to six-year cycles, allowing centres to adopt newer, more efficient technologies on a rolling basis.

### Lessons for the UK

This international comparison yields several observations relevant to UK policy:

- Long-term funding certainty is a precondition for effective investment in sustainability. Building modifications for waste heat recovery, power infrastructure upgrades, and renewable energy procurement contracts all require multi-year financial commitments that cannot be made under short funding cycles. The DOE and EuroHPC models provide this certainty explicitly.
- Rather than committing to a monolithic HPC procurement designed to remain static for five or six years, a more effective strategy is to plan incremental upgrades on an 18- to 24-month cadence, aligning refresh cycles with the rapid pace of advancement in hardware. This approach demands the underlying system architecture to be flexible enough to accommodate not just planned hardware swaps, but also changes in operating model. A system conceived with this philosophy treats flexibility as a first-class design requirement, ensuring that when the next opportunity or disruption arrives, the platform can evolve to meet it rather than constrain it.
- Sustainability requirements built into procurement produce measurably better outcomes. The EuroHPC and DOE systems have binding energy requirements as part of site selection and procurement criteria, whereas the UK has no equivalent.
- Skills and training ecosystems are treated as integral to HPC investment in most comparator countries. The EuroHPC programme includes dedicated funding for user training and centres of excellence, while the DOE’s ECP included substantial application development and porting support (especially before a new machine is deployed). The UK does fund skills and training activities, but there is a question of whether these are always directed at the most impactful areas. In particular, there is a strong case for greater investment in software porting and application development support to help users make effective use of new architectures.

## Sustainability and environmental impact

The growing scale and capability of HPC systems brings with it a significant and increasingly scrutinised environmental footprint, with modern supercomputers consuming substantial amounts of energy both for computation and for the cooling infrastructure required to manage the heat they generate. As the global push towards net zero accelerates, the HPC sector faces mounting pressure to demonstrate credible pathways to sustainability. This requires balancing the societal value of computational research against its environmental impact.

### Energy consumption and carbon emissions

In absolute terms, HPC systems are amongst the most energy-intensive computing infrastructures deployed by UK research institutions. Energy consumption is driven by processor and memory power draw, cooling systems, networking infrastructure, and storage systems. For ARCHER2, the operator has published a component-level apportionment of loaded power draw derived from a combination of on-system measurements and estimates supplied by the hardware vendor [1]:

- Compute nodes (5,860 nodes) draw 3,000 kW (∼85%)
- Slingshot interconnect (768 switches) draws 200 kW (∼5-6%)
- Cabinet overheads^5^ across 23 cabinets draw 200 kW (∼5-6%)
- Coolant distribution units (6 CDUs) draw 96 kW (∼3%)
- File systems (5 file systems) draw 40 kW (∼1%)

Of these, the per-component distribution across switches, cabinet overheads, coolant distribution units, and storage is vendor-estimated and should be read as indicative of relative magnitude rather than as a measured result. However, the aggregate is independently confirmed by facility telemetry. The mean power draw of the ARCHER2 compute cabinets (which enclose all compute nodes and interconnect switches, approximately 90% of the total power of the system) was measured from the chassis management infrastructure at 3,220 kW between December 2021 and April 2022, some 5% below the summed load estimate (3,400 kW), with the residual attributable to the system not operating at 100% load [1]. The non-compute fraction has also been measured directly. Controlled experiments filling a reserved 256-node cabinet with application benchmarks and comparing the metered cabinet energy against the sum of the on-node energy counters give overheads of 15.5% to 21% of the cabinet energy [30], consistent with the 15% increase the operator applies to the measured compute-node energy in its production energy and emissions reporting tooling [31]. The energy consumption measured in the facility for the service is reported quarterly by the operator [32]. The operator treats the overhead of the electrical and cooling infrastructure of the plant as typically less than 10% [31].

Optimised operations can reduce the power by more than 20%, corresponding to ≈0.7 MW lower than the theoretical power draw.

The Power Usage Effectiveness (PUE) ratio, which is the total facility energy divided by equipment energy, is the most widely used metric for data centre efficiency. A PUE of 1.0 is ideal and modern well-designed HPC facilities achieve PUEs of between 1.1 and 1.3. However, older air-cooled facilities may have PUE values of 1.5 or above. The ACF (Advanced Computing Facility) hosting ARCHER2 is estimated to have a PUE of around 1.1 [33].

In the UK, both ARCHER2 and Isambard-AI are powered by 100% renewable or zero-carbon electricity, meaning that carbon-aware scheduling based on grid carbon intensity would only offer limited direct benefit for either system. Both systems are holders of the REGO (Renewable Energy Guarantees of Origin) certificate in the UK, confirming that the electricity powering these supercomputing facilities is sourced from renewable energy. They also employ direct liquid cooling rather than traditional air cooling, significantly reducing cooling energy overhead.

In general, most HPC systems in the UK do not publish data on energy consumption and carbon emission. This absence makes it very challenging to assess the cumulative environmental footprint of publicly funded UK HPC at a national level. At the moment, there is no statutory obligation in the UK for data centres to report or maintain a specific energy efficiency status. In addition to the general energy efficiency standards (MEES) regulations, only voluntary schemes are available in this space [34].

### Embodied carbon

The carbon footprint of an HPC system is not limited to just operational energy. Indeed, *embodied carbon*, which corresponds to the emissions associated with the manufacturing, transport, and installation of hardware, represents a significant and largely unquantified fraction of the total lifecycle impact. For example, semiconductor fabrication is energy-intensive and involves complex global supply chains.

Embodied carbon is a fixed cost at the point of manufacture, and once a system is deployed, the operator cannot reduce it. However, it can be amortised over a longer operational lifetime. This therefore has direct relevance to decisions around hardware refresh cycles and decommissioning timelines because decommissioning a system after five years has a higher per-year embodied carbon cost than the same system which is instead operated for eight years.

However, this amortisation argument is only one side of the trade-off. Successive hardware generations typically deliver more work per unit of energy, so extending the life of an older system can increase operational emissions relative to a more efficient replacement. The decision is therefore best framed as a carbon payback problem: replacement incurs the embodied cost of the new system immediately and is justified only where the operational saving, integrated over the remaining useful life of both the incumbent and its successor, exceeds that cost.

The term that dominates depends principally on the intensity of carbon in the electricity supply. Following the analysis of [1], where carbon intensity is very low, lifetime emissions are dominated by embodied carbon and the most effective strategy is to extract the maximum output per node-hour as long as the system remains serviceable. Where carbon intensity is high, operational emissions dominate, and the payback period for a more efficient replacement is correspondingly shorter, so that earlier replacement may reduce total lifetime emissions. At intermediate intensities, the two contributions are comparable and the outcome is genuinely sensitive to the assumptions made.

Two further considerations should be taken into account. First, generational efficiency gains are not automatically realised: their benefit depends on whether the existing workload can exploit the new architecture, and substantial porting or re-optimisation effort may be required before headline efficiency improvements translate into improvements in delivered science per kWh. Meaningful operational savings are also available on existing hardware without replacement; on ARCHER2, configuration changes alone reduced the mean compute-cabinet power draw by 21% [1]. Second, efficiency gains per unit of work have historically been accompanied by increases in absolute system power, so replacement commonly raises, rather than lowers, the total energy demand and embodied footprint of a facility, even where it improves efficiency metrics.

Embodied carbon must be part of the conversation around hardware lifecycle decisions and is primarily a matter for manufacturers and funders rather than for HPC operators acting alone. The interaction between embodied carbon and operational carbon in different electricity environments is complex and is not adequately captured by simple offset frameworks. Determining the optimal system lifetime is a multi-parameter optimisation problem which is subject to diminishing returns. The carbon benefits of extending operational life must be weighed against factors such as the pace of technological change, improvements in energy efficiency of newer hardware, and evolving workload requirements. Ultimately, there is no single answer that applies universally.

### Hardware lifecycle and recycling

The end-of-life management of HPC hardware is poorly documented in the UK, and practices depend significantly on the machine’s ownership structure and a centre’s contractual obligations. HPE operates a formal asset upcycling and recycling service for its hardware, and previously Cray operated a Take-Back Programme for decommissioned supercomputers [35]. When providing hardware to the public sector, the manufacturer typically recovers the hardware at the end of the contract for refurbishment and materials recycling, for instance HPE will reclaim and recycle ARCHER2 when it is decommissioned. For owned equipment, the host institution is responsible for disposal through re-use, sale as second-hand equipment, or formal waste electrical and electronic equipment (WEEE) recycling channels.

ARCHER2 is scheduled to be decommissioned in November 2026, approximately five years after the service opened. Its predecessors ran for longer; for instance, ARCHER was operational for eight years (2014-2021) and HECToR for approximately seven years (2007-2014). However, these systems were intended to have shorter lifetimes and were granted explicit service extensions driven by the delayed delivery of their successors and the requirement to provide a continuous national service. By contrast, Japan’s K Computer and Fugaku both had planned operational lifetimes of approximately eight years. Whether a five-year operational lifetime for a supercomputer such as ARCHER2 is appropriate should be assessed with consideration given to energy efficiency trends over the system’s life, cost of continued operation versus replacement, embodied carbon amortisation and utilisation rates. No publicly available analysis has been published to this effect.

Hardware repurposing options for decommissioned HPC systems include continued use within the host institution, donation, or low-cost transfer to other research institutions, and, where technically and logistically feasible, redeployment to institutions in other countries. The last option requires a careful assessment of operational costs and determining whether it represents genuine value rather than export of difficult-to-manage equipment. Low-cost transfers tend to work best for mid-range cluster-style systems with modest infrastructure requirements, rather than bespoke flagship machines with specialised cooling and power demands.

In 2012, Los Alamos National Laboratory (LANL) transferred over 2,000 retired computers to Carnegie Mellon and the New Mexico Consortium, backed by National Science Foundation funding, to create the PRObE Centre, the world’s first large-scale facility dedicated to open systems software research [36]. Building on this, in 2017 LANL donated a further decommissioned supercomputer directly to the University of New Mexico, where it became the most powerful machine on campus. In both cases, the transferred hardware was over five years old [37].

In summary, there is a notable gap in transparency around the full lifecycle environmental impact of national HPC systems. The embodied carbon and end-of-life handling of hardware rarely receives the same attention as the operational energy consumption, despite having a significant environmental impact.

### The lack of sustainability metrics

The UK currently has no standardised framework for measuring, reporting, and benchmarking the environmental impact of publicly funded HPC systems. The absence of such a framework means that it is not possible to compare sustainability metrics of different UK systems on a like-for-like basis. Consequently, improvements or deteriorations in national-level sustainability performance cannot be tracked. Ultimately, such a lack of tracking means that funding bodies cannot make evidence-based decisions about the environmental value of investments, and the UK cannot demonstrate responsible public investment management in this area.

Metrics that could form part of any such framework include energy consumption per system (MWh/year and normalised per job or per CPU-hour), PUE, carbon intensity of the energy consumed, Water Usage Effectiveness (WUE) and hardware utilisation rate. An end-of-life disposal pathway should also be provided. It is important to note that individual metrics can be misleading in isolation, for instance a very low PUE at a facility powered by fossil fuels is worse from a carbon perspective than a higher PUE at a facility powered by renewables. Therefore, metrics must be reported in context. It should also be noted that the effectiveness of PUE is often undermined by the fact that PUE values are frequently measured under optimal conditions rather than being computed as an annualised average. An average better accounts for variations in workload intensity and seasonal weather fluctuations, giving a more realistic and representative picture of a facility’s true energy efficiency.

## Accountability, transparency, and return on public investment

Given that publicly funded HPC systems are supported by the tax payer, accountability is crucial. Such accountability requires that users can demonstrate the value of their compute allocations through research outputs, operators can demonstrate efficient and sustainable system management, and funders can compare the cost-effectiveness of, and return on investment on, different projects.

It is important to state clearly that the aim of enhanced accountability is not punitive. Transparency in reporting poor energy efficiency, high idle time, or suboptimal use should be understood as a mechanism to help identify where support, investment, or operational changes are needed. Crucially, this should not be viewed as a basis for penalising operators, institutions, or even users. The appropriate response to identified inefficiencies is a structured improvement process.

### Current reporting landscape

EPCC’s machines ARCHER2 and Cirrus publish regular usage statistics, including the number of users and projects, research domain breakdown, utilisation rates, uptime, and annual summaries of supported research outputs. For medium-sized systems in the UK, the reporting practice varies substantially. Some systems publish annual reports whereas others provide no systematic public data. EPSRC/UKRI requires end-of-programme reports, but these are not always publicly available and do not follow a standardised format.

For recent AI-dedicated investments, such as Isambard-AI and Dawn via the AIRR programme, no standardised public reporting requirements appear to have been established as a condition of investment. Finally, for the new National Compute Resources, the reporting requirements, if any, have not yet been publicly specified in any detail.

### Proposed accountability framework

This paper proposes that all publicly funded HPC systems above a defined investment threshold should be required to publish, on an annual basis, a standardised report covering:

- System utilisation: the proportion of available compute hours used, broken down by research domain and user type.
- Energy and sustainability: the total energy consumed (MWh), PUE (including the parameters for measuring PUE), carbon intensity of energy used, water usage, and progress against any sustainability targets.
- Research outputs: publications, datasets, and other outcomes attributed to the system, using a consistent attribution methodology.
- User satisfaction and support: results of user surveys, support ticket resolution metrics, and training provision data.
- Hardware lifecycle status: expected operational lifetime, decommission planning, and end-of-life disposal approach.

At a minimum, UKRI should make compliance with a standardised reporting framework a condition of all future funding awards for HPC systems. The time-consuming effort to develop, implement, and use a reporting framework should be a funded activity included in the system budget and would ultimately be the responsibility of the operator.

## Skills, training, and user support

### The skills gap

Running HPC workloads is far more challenging than it appears on the surface. Inadequate use of HPC resources through suboptimal parallel code and I/O patterns, lack of understanding of HPC systems, or inappropriate use of compute resources, represents a real but difficult-to-quantify waste of compute cycles and energy. This problem is particularly acute because the majority of HPC users are domain scientists whose expertise lies in their research field, not in systems programming or HPC infrastructure.

Poor practices such as over-allocating nodes, failing to checkpoint long-running jobs, or submitting poorly tuned workloads can monopolise shared infrastructure and increase collective waste across an HPC facility. HPC users do not need to become systems experts, but it is important for them to develop a working understanding of how their job configurations impact resource consumption and what constitutes responsible use of a shared system. Better-informed users make better job submissions and that benefits everyone on a shared HPC system.

The scale of the problem is difficult to assess on a single system, let alone at a national level. This is because it depends on the distribution of coding expertise across the user community, the degree to which application support is available, and the nature of workloads being run. However, the existence of dedicated experts, training programmes, tutorials, specific support on HPC systems demonstrates that the problem is recognised and that targeted support produces measurable efficiency improvements.

### Current training provision

The UK research community has recognised these challenges and responded with a growing ecosystem of initiatives aimed at improving HPC training, skills development, and sustainable computing practices. The ARCHER2 Embedded CSE (eCSE) programme represents the most substantial investment in HPC software development and skills in the UK to date. Funding dozens of projects across more than ten calls between 2020 and 2024, this programme has enabled the research community to develop, optimise and maintain scientific software for ARCHER2[13]. More recently, the £129 million UKRI Digital Research Infrastructure (DRI) programme has funded a range of projects. These include dRTP Skills [38] which has worked to connect training opportunities with roles and people, making the ecosystem more transparent and easier to navigate for digital RTPs. DisCouRSE [39], also part of the DRI programme, focuses on developing leadership and career pathways across all digital research technical professional roles. CAKE [40] acts as a central hub for both the DRI programme and wider UK large-scale computing to bring these communities together and share best practices, tools, and resources.

On the sustainability front, NetDRIVE [41] brings together creative thinking from across UKRI to build a common vision for a sustainable DRI and incite a transition to sustainable working practices. SHAREing [42] is a UK-wide initiative focused specifically on performance analysis, optimisation, and sustainable computing practices across research institutions. The Institute of Research Software (formally the Software Sustainability Institute)’s Green DiSC programme [43] offers the first open-access certification scheme providing a roadmap for research groups and institutions who want to tackle the environmental impacts of their computing activities. The Green Research Software Engineer Special Interest Group [44] curates a wealth of resources, from free courses on sustainable computing to HPC-specific carbon footprint tools. Together, these initiatives reflect a broad and coordinated effort across the UK to raise both the technical literacy and the environmental awareness of the research computing community.

### Recommendations for skills development

The gap identified in Sect. 6.1, between the computational literacy domain scientists possess and the expertise effective HPC use actually requires [45], has a structural cause as well as an educational one. HPC work is typically funded indirectly within application domain grants rather than supported through dedicated mechanisms.

Making HPC training mandatory for researchers who rely on HPC resources is a logical step that several funding bodies and facilities are beginning to consider. ARCHER2 already incentivises users to participate in training as part of its service offering, setting a precedent that other HPC facilities in the UK have not consistently followed.

A stronger policy position, for instance, making completion of relevant training a condition of grant funding where HPC resources are involved, or embedding training requirements into facility access agreements, would help establish a more level playing field and reduce the waste that stems from poorly configured or inefficiently run jobs. Indeed, in 2013 DiRAC rolled out a mandatory in person *driving test* across its user base, requiring all users to complete a basic test that focused on key skills required to use the machines. This was discontinued, most likely due to the administrative burden of having to administer the tests, but the optional ARCHER2 *driving test* demonstrates that it is possible to provide this in a more convenient form.

On the question of what is missing from the current landscape, several gaps remain. Although some funding is directed towards HPC training, the current model which is largely centred on courses and workshops, has inherent limitations. Researchers may attend a session but struggle to apply their new skills in their daily practice without ongoing follow-up and support. A more effective approach would be to couple training investment with funded software porting and development activities, carried out by RTPs who are themselves developing expertise at the same time. This learn-by-doing model ensures that skills are honed through sustained, hands-on work on real codebases, rather than being lost after a one-off classroom experience. The community would also benefit from a more unified and accessible catalogue of existing training materials, which currently sit amongst various initiatives operating in relative isolation.

Furthermore, although there is a reasonable body of material covering general HPC usage and software sustainability, training specifically targeted at application scientists who need to understand the resource implications of their workflows is limited. Similarly, training on how to benchmark and profile code, and make informed decisions about resource requests is not yet systematically available across the UK HPC ecosystem.

Another important consideration is around the best use of AI-assisted coding tools that offer a potentially transformative role in HPC software development. Potential uses include accelerating the porting of legacy codes to new architectures, and suggesting performance optimisations that would be time-consuming to identify manually. However, realising this potential will require skilled RTPs and training provision to critically evaluate and guide AI-generated suggestions in the context of scientific applications.

Finally, funders *must* start recognising the skills involved in operating, using, and developing algorithms, software, and libraries for HPC systems as a specialist domain. The *science of supercomputing* requires expertise that is distinct from traditional science subjects and only by recognising this will it be possible to develop the necessary skills across user communities and ensure that HPC resources are used as efficiently as possible.

## AI investment and HPC: a strategic discussion

### The UK government’s current approach

Since approximately 2022, the UK government has pursued a dual-track investment strategy in compute infrastructure. This has involved an extremely difficult continuation of general-purpose HPC investment, most significantly the £750 M for ARCHER2’s successor, alongside targeted AI compute investments resulting in Isambard-AI, Dawn, and the AIRR programme [9]. The logic is that AI workloads may have different hardware requirements from traditional simulation (non-AI) workloads, and that the two should be provisioned separately. There is some technical justification behind this; however, many non-AI applications can leverage accelerator hardware such as GPUs. Such a dual-track strategy risks fragmentation, reduced scheduling flexibility on both tracks, duplication of operational overhead, and uneven sustainability requirements across the two tracks. As noted in Sect. 1.1, whether reduced scheduling flexibility translates into measurably lower achieved utilisation cannot be determined from public data, since operational statistics for the UK’s AI-dedicated systems are not currently published.

Furthermore, AI training, AI inference, tightly coupled simulation, and data-intensive workflows have materially different requirements, and any argument for integration that ignores this is not credible. The question is whether those differences align with the AI/non-AI boundary along which UK investment has been partitioned. We argue that they may not.

It is worth stating that the dual-track approach has a coherent policy rationale that goes beyond workload characteristics. Sovereign AI compute capability is being pursued by most comparable economies as a matter of industrial strategy and national security, not solely of research provision; the AI Opportunities Action Plan [10] and the June 2026 AI Hardware Plan [11] explicitly state that the objective includes growing a domestic chip and hardware sector, attracting private investment, and retaining strategic capability onshore. Demand for AI compute is also commercially time-sensitive in a way that simulation demand is not, and a dedicated procurement route allows government to move at a pace that general research infrastructure cycles do not readily accommodate. These are legitimate objectives, and we do not argue that public investment in AI compute is misdirected.

Our concern is narrower. Objectives of this kind justify additional investment in compute; they do not by themselves justify partitioning the national HPC infrastructure along an AI/non-AI boundary, restricting access to those systems by application domain, or exempting them from the reporting requirements applied elsewhere. A system procured to build sovereign capability can be operated as a shared national resource with standardised utilisation and energy reporting, and would demonstrate the value of that investment more convincingly for being so.

### International approaches to AI and HPC integration

The relationship between AI infrastructure and general HPC varies across comparable countries. EuroHPC systems are increasingly heterogeneous, incorporating both CPU and GPU nodes within single systems. The DOE’s Frontier and Aurora systems similarly provide GPU-heavy architectures capable of supporting both simulation and AI workloads. Moreover, the NAIRR initiative in the USA is designed to integrate AI and HPC resources under a single access framework. The common international trend is towards integrated, heterogeneous systems rather than separate AI and HPC investments. This is in contrast to the UK’s dual-track approach which risks creating siloed infrastructure with separate user communities, separate training requirements, and separate accountability structures. Furthermore, it means that, in the UK, large-scale non-AI workloads will be left without any viable computing platform during the gap between ARCHER2’s decommissioning and the new national supercomputing service coming online. Even in the best case scenario it is likely that this gap will be over a year long.

### Risks of conflating AI and HPC

There are also risks in the opposite direction, namely treating AI as a competitor with HPC. Such public discourse has led to investment decisions that prioritise AI workloads at the expense of the broader research community dependent on traditional simulation workloads. Climate modelling, materials science, structural biology, and engineering simulation in the UK are currently mainly reliant on CPU-based systems, but many simulation software have been ported to leverage GPUs at scale in the same way as AI. If the national investment strategy implicitly treats GPU computing as the definition of HPC, these communities need significant support to leverage GPU acceleration. Two recent examples of software programmes in the UK are ExCALIBUR [14] and the eCSE-GPU programme, both of which aimed to progress the software ecosystem towards being exascale-ready, but have suffered from a lack of long-term investment.

Our position is therefore not that all workloads have the same requirements, but that AI and non-AI workloads should not be used to make decision for the procurement of a new HPC system. A procurement boundary drawn along that line will systematically misallocate capability, because it partitions hardware on a criterion that does not track the technical characteristics the partitioning is intended to serve. A system procured for AI and restricted to AI may be well matched to training and poorly matched to inference; a CPU-only national service excludes GPU-ported simulation codes that would run well on supercomputers dedicated to AI. Policy and investment frameworks should ensure that the full breadth of the research community’s compute needs is addressed coherently.

## Recommendations

The following recommendations are directed at three distinct but interdependent groups: 1) government and policymakers who set the strategic and regulatory framework, 2) the HPC community responsible for operating and developing systems, and 3) funders who shape what gets built and supported. Taken together, they represent a coherent set of actions that would move the UK towards a more accountable, sustainable, and effective HPC ecosystem. None of these recommendations requires new mechanisms, and most are based on existing frameworks, initiatives, and good practice that are already present in parts of the landscape, but have not yet been applied consistently or at scale.

### For government and policymakers

The government plays a fundamental role in setting the conditions under which HPC investment is made, governed, and evaluated. The recommendations that follow focus on establishing the strategic coherence and accountability structures that are currently absent, and without which individual facility-level efforts will remain fragmented. A clearer policy framework would also give the HPC community greater certainty and a more consistent basis on which to plan long-term infrastructure investments.

- Adopt a long-term coherent and rolling national HPC strategy. This should be published and reviewed on a ten-year horizon, with sustainability requirements built in from the outset. The recently published UK Compute Roadmap is a good starting point [46].
- Introduce mandatory standardised public sustainability reporting for all publicly funded HPC systems that receive investment above a defined threshold, as a condition of funding.
- Introduce accountability requirements for data use, research output tracking, and cost-effectiveness metrics, aligned with the framework in Sect. 5.2.
- Establish transparent, evidence-based criteria for hardware lifecycle decisions, with embodied carbon and operational efficiency as explicit factors.
- Ensure that all new investments in national compute resources include binding sustainability and reporting obligations from the point of contract.
- Review the dual-track AI/HPC investment approach for coherence, with a view to establishing integrated governance, access, and reporting frameworks across both tracks.

### For the HPC community

HPC centres and the broader technical community are best placed to define what good practice looks like in operation and to develop the tools and standards that make sustainability measurable and comparable across systems (this would require adequate funding). The recommendations here are intended to move the community from isolated good intentions to shared, auditable commitments. Many of the building blocks already exist in a nascent form, through initiatives such as those described in the previous sections, but these need to be consolidated, standardised, and applied universally rather than selectively.

- Develop and adopt standardised sustainability metrics, in collaboration with UKRI and with reference to international frameworks (e.g. the Green Grid, ISO 30134 series).
- Invest in user training and RTP support proportionate to capital investment in hardware where training and support budgets should be explicitly costed in all funding applications.
- Publish annual reports for all HPC centres with comprehensive usage, impact, and sustainability data, using a consistent format.
- Establish and publicise best-practice guidelines for end-of-life management of hardware, including recycling, repurposing, and disposal pathways.

### For funders (including, but not limited to, UKRI)

Funders have a uniquely influential position because the conditions they attach to grants and infrastructure investments shape behaviour across the entire ecosystem. The following recommendations invite funders to use that influence more deliberately, not simply to require sustainability plans as a compliance exercise, but to treat training, skills, and long-term operational sustainability as integral components of what it means to fund HPC responsibly.

- Require sustainability and accountability plans as a mandatory component of all HPC funding investments, with evaluation criteria applied consistently.
- Require explicit consideration and reporting of energy use for HPC-enabled research projects.
- Fund research into embodied carbon measurement and attribution methodology for HPC infrastructure, in collaboration with manufacturers and international partners.
- Formally recognise HPC and supercomputing as a distinct scientific discipline, with its own career pathways, peer review expertise, and funding categories. UKRI and other funders must move beyond classifying supercomputing under a generic "IT" provision and instead acknowledge the *science of supercomputing* as a specialist domain requiring dedicated recognition, investment, and professional standing.
- Support skills development programmes tied to HPC access, including funding for RTP posts and training provision as an integral part of system operation grants.
- Develop long-term funding mechanisms that provide operational certainty over at least ten years, enabling the infrastructure investments in cooling, power, and buildings that sustainable HPC requires.

## Conclusion

The UK has made substantial public investment in HPC infrastructure, and that infrastructure is genuinely central to the country’s research capability, as demonstrated by a significant return on investment (a factor of eight for every £ spent on ARCHER2 [3]). However, investment in hardware alone is insufficient, and the absence of a standardised sustainability framework, the inconsistency of public accountability reporting, structural issues in procurement governance, and the lack of long-term funding certainty represent systemic weaknesses that diminish the return on public investment and create unnecessary environmental risk.

Standardised operational reporting, sustainability criteria in procurement, and long-horizon funding commitments all exist in some form within the UK landscape or within the comparator programmes examined in Sect. 3; what is absent is their consistent application as conditions of public investment.

With UK net zero targets requiring economy-wide reductions in emissions, and with HPC systems consuming megawatts of power continuously over multi-year lifetimes, the environmental credentials of public compute investments are a matter of legitimate public interest. The path to sustainable HPC in the UK is well-defined; what is required is the policy framework and institutional commitment to follow it.

## Data Availability

No datasets were generated or analysed during the current study.

## Footnotes

1 Enterprise IT infrastructure refers to the hardware, software, networks, and services that large organisations use to run their day-to-day business operations, such as servers, storage systems, databases, networking equipment, and the applications that support functions such as finance, HR, communications, and customer management.

2 UKRI is the umbrella body in the UK that brings together seven research councils, Innovate UK, and Research England to oversee and distribute UK government funding for research, innovation, and postgraduate training across all disciplines.

3 Mary Coombs is now part of the NCR programme, see next section.

4 The HPC TOP500 is a ranking of the 500 most powerful supercomputers in the world, measured twice yearly. It is based on the LINPACK test, a benchmark that measures HPC computing power by solving dense systems of linear equations. Not all large-scale systems necessarily enter a submission to the TOP500 list.

5 Cabinet overheads comprise the per-cabinet infrastructure load, principally power conversion losses, internal air movement, and management hardware, which is largely independent of node utilisation.
